# Prevalence and progression of visual impairment in patients newly diagnosed with clinical type 2 diabetes: a 6-year follow up study

**DOI:** 10.1186/1471-2458-11-80

**Published:** 2011-02-04

**Authors:** Niels de Fine Olivarius, Volkert Siersma, Gitte Juul Almind, Niels Vesti Nielsen

**Affiliations:** 1 The Research Unit for General Practice and Section of General Practice, Department of Public Health, University of Copenhagen, Copenhagen, Denmark; 2 University Eye Clinic, Rigshospitalet, Copenhagen, Denmark

## Abstract

**Background:**

Many diabetic patients fear visual loss as the worst consequence of diabetes. In most studies the main eye pathology is assigned as the cause of visual impairment. This study analysed a broad range of possible ocular and non-ocular predictors of visual impairment prospectively in patients newly diagnosed with clinical type 2 diabetes.

**Methods:**

Data were from a population-based cohort of 1,241 persons newly diagnosed with clinical, often symptomatic type 2 diabetes aged ≥ 40 years. After 6 years, 807 patients were followed up. Standard eye examinations were done by practising ophthalmologists.

**Results:**

At diabetes diagnosis median age was 65.5 years. Over 6 years, the prevalence of blindness (visual acuity of best seeing eye ≤ 0.1) rose from 0.9% (11/1,241) to 2.4% (19/807) and the prevalence of moderate visual impairment (> 0.1; < 0.5) rose from 5.4% (67/1,241) to 6.7% (54/807). The incidence (95% confidence interval) of blindness was 40.2 (25.3-63.8) per 10,000 patient-years. Baseline predictors of level of visual acuity (age, age-related macular degeneration (AMD), cataract, living alone, low self-rated health, and sedentary life-style) and speed of continued visual loss (age, AMD, diabetic retinopathy (DR), cataract, living alone, and high fasting triglycerides) were identified.

**Conclusions:**

In a comprehensive assessment of predictors of visual impairment, even in a health care system allowing self-referral to free eye examinations, treatable eye pathologies such as DR and cataract emerge together with age as the most notable predictors of continued visual loss after diabetes diagnosis. Our results underline the importance of eliminating barriers to efficient eye care by increasing patients' and primary care practitioners' awareness of the necessity of regular eye examinations and timely surgical treatment.

## Background

In Europe and the United States severe visual impairment may be 2-3 times more common among people with diabetes than in the general population [1,2], but this difference decreases with age [2]. Diabetic retinopathy (DR) is regarded as the cause of blindness in 5-15% of the blind in the general population [3-5] and in 30-50% of blind type 2 diabetic patients [6,7]. There are, however, huge regional differences in presumed causes of blindness across the world [8]. DR is considered the leading cause of blindness among people of working age in many countries [3-5], while age-related macular degeneration (AMD) is considered the leading cause in people over 65 years [3-5,9].

Diabetes with even mildly to moderately impaired sight has a negative impact on perceived quality of life and psychosocial functioning giving rise to feelings of vulnerability, worries about the future and loss of independence and mobility [10-12]. A sizeable proportion of type 2 diabetic patients fear visual loss intensely [13] and consider loss of vision the worst complication of diabetes [10]. For the health practitioner visual acuity is a ubiquitous and handy measure of visual function, but visual acuity is not a suitable measure of future visual loss as the sight-threatening eye pathologies often are present for many years before vision begins to decline as a result of these pathologies.

In the history of diabetes treatment, the development of diabetic retinopathy has been included in the outcome of numerous clinical trials though prevention of visual loss is the ultimate target for the patients. While the ocular predictors of future visual loss in diabetic patients are well-described [14-19], a comprehensive prospective study of ocular and non-ocular predictors of long-term changes in measured visual acuity has not been published before [14,16,19-23].

Our main aim was to study a broad range of predictors of vision loss in a population-based sample of patients newly diagnosed with type 2 diabetes and observed for 6 years.

## Methods

### Study population

**Figure 1:**
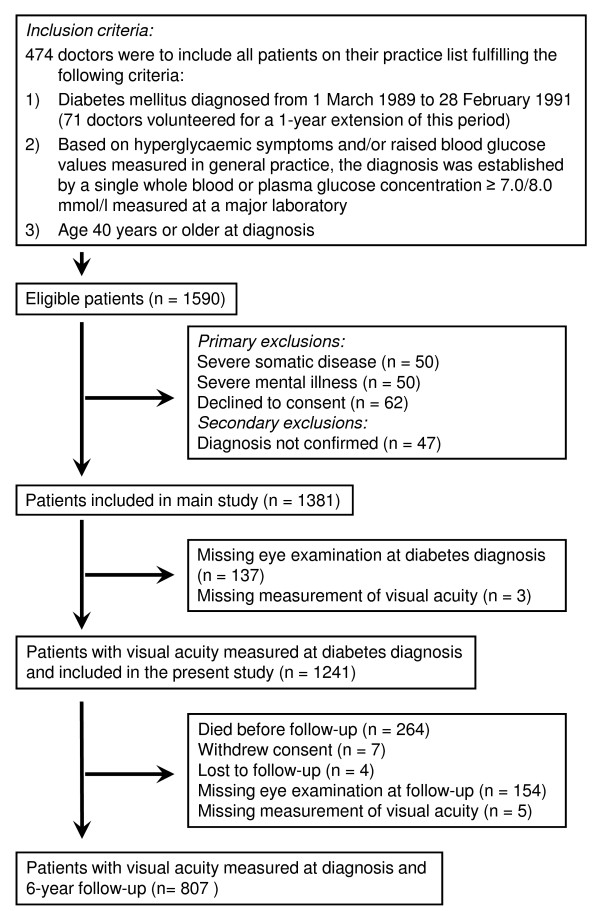
**Flow of participants through study**.

In the Danish Diabetes Care in General Practice study [24], 474 general practitioners agreed to include all subjects with newly diagnosed diabetes on their practice list (Figure 1). The 140 patients without a measurement of visual acuity at diagnosis did not differ from the 1,241 patients who were included in the present study regarding age (*p *= 0.36), sex (*p *= 0.31) and diagnostic plasma glucose (*p *= 0.81). At 6-year follow up, the 159 non-censored patients without information about visual acuity (Figure 1) did not differ from the 807 re-examined patients with regard to age (*p *= 0.23), sex (*p *= 0.82), diagnostic plasma glucose (*p *= 0.43), prevalence of DR at diagnosis (3.2% (5/158) vs. 4.4% (35/800), *p *= 0.49) and prevalence of moderately impaired vision or worse at diagnosis (7.0% (11/159) vs. 3.3% (27/807), *p *= 0.10). A small number started insulin treatment within 180 days of diagnosis, so 97.6% of the 1381 patients who had started in the study were considered to have type 2 diabetes [24]. Informed consent was obtained from all participants, and the protocol was approved by the ethics committee for Copenhagen and Frederiksberg.

### Ophthalmologic examination

Immediately after diabetes diagnosis, the general practitioner referred the patient to a practising ophthalmologist who did a standard eye examination. The result of the funduscopy was recorded by the ophthalmologist in a multiple choice question with six response categories: no diabetic lesions, microaneurysms only, background retinopathy without or with maculopathy, and proliferative retinopathy without or with new vessels on the optic disc. In open-ended questions information about other retinal pathologies, glaucoma, and eye operations was given, and the presence of cataract was indicated in a closed question. Information about glaucoma was also given as answers to open-ended questions about reasons for impaired vision. Visual acuity with best correction was estimated with an optotype chart, usually a Snellen chart, and given for each eye as the Snellen ratio at 6 metres or 20 feet or as decimal acuity, but all measurements were converted to decimal acuity and logMAR [25]. The time (interquartile range, IQR) from diabetes diagnosis until the first eye examination was 45 (24-83) days. A total of 164 ophthalmologists participated, and 68.5% (553/807) of patients were seen by the same ophthalmologist at first and last eye examination. The eye with best visual acuity was used for evaluation of retinal pathology.

### Assessments

The doctors recorded the following information about the patient: height and weight without shoes and outer garments; blood pressure and heart rate by routine methods after a 10-minute rest in a sitting position; sense of touch of cotton wool and pin prick on both feet; presence of dorsalis pedis or posterior tibial pulse on both feet; presence of patellar reflexes; history of myocardial infarction and/or stroke causing hospitalization; and amputation of any part of leg or foot before or at the time of diabetes diagnosis.

In questionnaires, patients gave information about whether they lived alone, their education, familial disposition to diabetes, smoking habits, leisure time physical activity, angina pectoris, intermittent claudication, and global self-rated health.

### Definitions

The decimal acuity of patients' best seeing eye was used to categorise patients according to usual clinical and administrative practice in Denmark: normal vision (≥ 0.5), moderate visual impairment (< 0.5 and > 0.1) or blindness (≤ 0.1). Cardiovascular disease was defined as history of myocardial infarction and/or history of stroke and/or angina pectoris and/or intermittent claudication and/or absent arterial pulses on both feet and/or amputation on the lower extremities. Peripheral neuropathy was defined as lack of a sense of pin prick and/or touch of cotton wool on at least one foot and/or absent patellar reflex on at least one knee.

### Assays

Laboratory analyses were centralized and quality controlled. Fasting blood samples were analysed at Odense University Hospital. Throughout the study, haemoglobin A1c (HbA_1c_) was determined by the same ion-exchange, high-performance liquid chromatography method. Samples from 100 blood donors (age 20-80 years, 33 men, 67 women) were analysed, and the reference interval (mean ± 2SD) was calculated to be 5.4-7.4%. Quality assurance was obtained with commercial control preparations from Bio-Rad. In October-December 1995, the mean (SD) of low (n = 24) and high (n = 29) control samples were 6.7 (0.31)% and 10.4 (0.63)%, respectively, resulting in coefficients of variation (CV = SD × 100/mean) of 4.6% and 6.0%. Serum total cholesterols were measured enzymatically with cholesterol esterase-cholesterol oxidase-peroxidase reagent, and fasting serum triglycerides was determined enzymatically with a lipase-glycerolkinase-glycerol-3-phosphate oxidase-peroxidase reagent. Serum creatinine was determined by the Jaffe reaction. In this nationwide study a freshly voided morning urine sample was used instead of timed urine collection to determine renal involvement. Urinary albumin concentration was measured at Århus University Hospital by a polyethylene glycol radioimmunoassay. Spot urinary albumin concentration is of value in predicting both progression of renal involvement and increased mortality, as is albumin excretion rate [26].

### Statistical analysis

The influence of baseline characteristics on change in visual acuity over 6 years was investigated in linear mixed models [27] using all available measurements of visual acuity from the best seeing eye. For each of the characteristics two models were constructed: one model where the change in visual acuity was described by a linear time trend (intercept and slope for the average change in visual acuity during the study) that differed for each of the categories of the characteristic, and a second model where the time trend additionally depended on age and sex in a multivariable regression fashion. Within-patient correlation of the observations was modelled by a patient random effect. The effect of the characteristic on changes in visual acuity was summarized with the fixed effect intercept and slope for each of the covariate classes from the first model (i.e. excluding age and sex). Differences between the covariate categories in intercept and slope in the two models were tested with the appropriate F-tests. In the 5.8% (72/1241) of patients who had an eye operation in the best seeing eye during follow up, measurements of visual acuity made after the operation were excluded from the analyses.

For the patients who were not moderately visually impaired or blind at baseline, incidence rates for these conditions were estimated as the ratio between (1) the number of patients with moderately impaired vision/blindness at follow up and (2) the sum of follow up times. The follow up times were halved for patients who became moderately visually impaired or blind during follow up, and confidence intervals were calculated assuming Poisson-distributed occurrences of vision impairment. Simple comparisons were made with Kruskal-Wallis tests and χ^2^-tests. To account for multiple statistical testing, we applied the method of Benjamini-Hochberg [28] on all the four columns of p-values in Table 1, i.e. 4 × 28 = 112 p-values, and found that a significance level of 0.0179 was to be used in order to control the false discovery rate at 5%. Accordingly, the nominal level of statistical significance was chosen to be *p *< 0.05, except in the mixed models in Table 1, where it was *p *< 0.01.

**Table 1:** Average changes in visual acuity during the first 6 years after diabetes diagnosis according to baseline patient characteristics

					Test of difference in level of logMAR at diabetes diagnosis ^a^		Test of difference in change of logMAR after diabetes diagnosis ^b^			
							
Characteristic at diagnosis	Category	*n*	Average logMAR at diabetes diagnosis	Average yearly change in logMAR after diabetes diagnosis	Un-adjusted *p*	Age and sex adjusted *p*	Un-adjusted *p*	Age and sex adjusted *p*	Median decimal acuity at diabetes diagnosis	Median decimal acuity 5.6 years after diabetes diagnosis
**Total**		**1241**	**-0.24**	**-0.0250**					**1.00 (0.80-1.00)**	**0.80 (0.67-1.00)**
										
Sex	Female	588	-0.30	-0.0343	0.001	0.070	0.0006	0.017	0.90 (0.67-1.00)	0.80 (0.60-1.00)
	Male	653	-0.19	-0.0160					1.00 (0.80-1.00)	1.00 (0.67-1.00)
										
Age (years)	40- < 60	430	-0.05	-0.0068	< 0.0001	< 0.0001	< 0.0001	< 0.0001	1.00 (1.00-1.00)	1.00 (0.80-1.00)
	60- < 70	377	-0.18	-0.0125					1.00 (0.80-1.00)	0.80 (0.67-1.00)
	≥ 70	434	-0.47	-0.0769					0.70 (0.60-0.95)	0.60 (0.40-0.80)
										
Living alone	Yes	384	-0.39	-0.0527	< 0.0001	0.0009	< 0.0001	0.002	0.80 (0.67-1.00)	0.67 (0.50-0.90)
	No	835	-0.17	-0.0158					1.00 (0.80-1.00)	1.00 (0.70-1.00)
										
Education	Basic	935	-0.26	-0.0293	0.006	0.67	0.008	0.30	1.00 (0.70-1.00)	0.80 (0.60-1.00)
	Higher	252	-0.15	-0.0114					1.00 (0.90-1.00)	1.00 (0.70-1.00)
										
Residence	Countryside	486	-0.26	-0.0269	0.41	0.98	0.56	0.60	1.00 (0.80-1.00)	0.80 (0.63-1.00)
	Larger towns	430	-0.24	-0.0211					1.00 (0.80-1.00)	0.80 (0.63-1.00)
	Copenhagen area	273	-0.20	-0.0211					1.00 (0.80-1.00)	0.80 (0.67-1.00)
										
Familial disposition to DM	No	659	-0.25	-0.0314	0.58	0.76	0.041	0.028	1.00 (0.75-1.00)	0.80 (0.63-1.00)
	Yes	466	-0.23	-0.0195					1.00 (0.80-1.00)	0.90 (0.67-1.00)
										
Diagnostic plasma glucose (mmol/l)	< 10	220	-0.23	-0.0227	0.93	0.47	0.36	0.063	1.00 (0.80-1.00)	0.80 (0.63-1.00)
	10-< 17	706	-0.24	-0.0281					1.00 (0.80-1.00)	0.80 (0.67-1.00)
	≥ 17	315	-0.24	-0.0193					1.00 (0.80-1.00)	0.80 (0.60-1.00)
										
Haemoglobin A_1c _(%) ^c^	< 9	298	-0.20	-0.0239	0.45	0.35	0.083	0.071	1.00 (0.80-1.00)	0.80 (0.67-1.00)
	9- < 11	347	-0.24	-0.0347					1.00 (0.80-1.00)	0.90 (0.67-1.00)
	≥ 11	396	-0.25	-0.0195					1.00 (0.70-1.00)	0.80 (0.60-1.00)
										
Total cholesterol (mmol/l)	< 6	495	-0.27	-0.0190	0.22	0.55	0.20	0.076	1.00 (0.70-1.00)	0.80 (0.60-1.00)
	6-< 7	366	-0.24	-0.0297					1.00 (0.80-1.00)	0.90 (0.67-1.00)
	≥ 7	358	-0.20	-0.0277					1.00 (0.80-1.00)	0.80 (0.67-1.00)
										
Fasting triglycerides (mmol/l)	< 2	616	-0.24	-0.0201	0.89	0.75	0.022	0.0008	1.00 (0.80-1.00)	0.80 (0.67-1.00)
	2- < 3	320	-0.25	-0.0227					1.00 (0.80-1.00)	0.80 (0.60-1.00)
	≥ 3	279	-0.23	-0.0388					1.00 (0.80-1.00)	0.80 (0.67-1.00)
										
Urinary albumin (mg/l)	< 15	703	-0.19	-0.0205	0.004	0.015	0.038	0.13	1.00 (0.80-1.00)	0.90 (0.67-1.00)
	15-< 200	437	-0.31	-0.0347					1.00 (0.70-1.00)	0.80 (0.60-1.00)
	≥ 200	59	-0.21	-0.0172					1.00 (0.80-1.00)	0.80 (0.60-1.00)
										
Serum creatinine (μmol/l)	< 130	1166	-0.23	-0.0238	0.012	0.33	0.012	0.062	1.00 (0.80-1.00)	0.80 (0.67-1.00)
	≥ 130	54	-0.43	-0.0634					0.80 (0.67-1.00)	0.75 (0.50-1.00)
										
Height - men (cm)	< 165	69	-0.30	-0.0273	0.11	0.58	0.53	0.96	0.95 (0.70-1.00)	0.80 (0.60-1.00)
	165- < 180	470	-0.18	-0.0156					1.00 (0.80-1.00)	1.00 (0.67-1.00)
	≥ 180	113	-0.14	-0.0135					1.00 (0.90-1.00)	1.00 (0.80-1.00)
										
Height - women (cm)	< 150	19	-0.47	-0.0587	0.050	0.47	0.12	0.91	0.70 (0.70-0.80)	0.60 (0.50-0.80)
	150- < 165	415	-0.32	-0.0390					0.80 (0.67-1.00)	0.80 (0.50-1.00)
	≥ 165	154	-0.20	-0.0203					1.00 (0.80-1.00)	0.90 (0.70-1.00)
										
Systolic blood pressure (mm Hg)	< 140	366	-0.18	-0.0174	0.023	0.78	0.036	0.63	1.00 (0.80-1.00)	1.00 (0.67-1.00)
	140-< 160	414	-0.25	-0.0223					1.00 (0.70-1.00)	0.80 (0.63-1.00)
	≥ 160	459	-0.28	-0.0338					1.00 (0.75-1.00)	0.80 (0.62-1.00)
										
Resting heart rate (bpm)	< 80	754	-0.23	-0.0208	0.17	0.09	0.040	0.066	1.00 (0.80-1.00)	0.80 (0.67-1.00)
	80- < 90	333	-0.23	-0.0361					1.00 (0.70-1.00)	0.80 (0.60-1.00)
	≥ 90	149	-0.32	-0.0219					1.00 (0.67-1.00)	0.80 (0.63-1.00)
										
Cardiovascular disease	No	849	-0.20	-0.0224	< 0.0001	0.045	0.035	0.55	1.00 (0.80-1.00)	0.90 (0.67-1.00)
	Yes	367	-0.34	-0.0357					0.90 (0.67-1.00)	0.80 (0.50-1.00)
										
Peripheral neuropathy	No	992	-0.22	-0.0218	0.013	0.14	0.013	0.016	1.00 (0.80-1.00)	0.90 (0.67-1.00)
	Yes	232	-0.32	-0.0392					0.90 (0.67-1.00)	0.80 (0.60-1.00)
										
Self-rated health	Excellent or good	557	-0.18	-0.0200	0.0006	0.003	0.043	0.13	1.00 (0.80-1.00)	0.90 (0.70-1.00)
	Fair, poor or very poor	663	-0.29	-0.0309					0.90 (0.70-1.00)	0.80 (0.60-1.00)
										
Smoking	Never	372	-0.33	-0.0369	0.0007	0.32	0.009	0.50	0.90 (0.67-1.00)	0.80 (0.60-1.00)
	Former	431	-0.22	-0.0206					1.00 (0.80-1.00)	0.80 (0.63-1.00)
	Current	414	-0.18	-0.0186					1.00 (0.80-1.00)	1.00 (0.70-1.00)
										
Physical activity	Sedentary	323	-0.39	-0.0364	< 0.0001	< 0.0001	0.035	0.20	0.80 (0.60-1.00)	0.70 (0.50-1.00)
	Non-sedentary	893	-0.19	-0.0227					1.00 (0.80-1.00)	0.90 (0.67-1.00)
										
Weight - men (kg)	< 80	200	-0.24	-0.0172	0.19	0.85	0.64	0.81	1.00 (0.80-1.00)	0.80 (0.67-1.00)
	80- < 90	196	-0.18	-0.0204					1.00 (0.80-1.00)	1.00 (0.80-1.00)
	≥ 90	255	-0.16	-0.0130					1.00 (0.80-1.00)	1.00 (0.70-1.00)
										
Weight - women (kg)	< 70	200	-0.39	-0.0485	0.008	0.21	0.040	0.068	0.80 (0.67-1.00)	0.70 (0.50-1.00)
	70-< 80	157	-0.29	-0.0208					0.90 (0.67-1.00)	0.80 (0.60-1.00)
	≥ 80	231	-0.21	-0.0319					1.00 (0.80-1.00)	0.80 (0.63-1.00)
										
Diabetic retinopathy	None	1186	-0.23	-0.0243	0.040	0.012	< 0.0001	0.0005	1.00 (0.80-1.00)	0.80 (0.67-1.00)
	Microaneurysms only	14	-0.54	0.0174					1.00 (0.90-1.00)	0.85 (0.80-1.00)
	Further retinopathy	38	-0.38	-0.1164					0.80 (0.63-1.00)	0.60 (0.50-0.80)
										
Age-related macular degeneration	No	1087	-0.18	-0.0170	< 0.0001	< 0.0001	< 0.0001	< 0.0001	1.00 (0.80-1.00)	0.90 (0.67-1.00)
	Yes	154	-0.67	-0.1000					0.67 (0.50-0.80)	0.50 (0.40-0.67)
										
Cataract	No	889	-0.14	-0.0145	< 0.0001	< 0.0001	< 0.0001	0.0004	1.00 (0.80-1.00)	1.00 (0.70-1.00)
	Yes	337	-0.51	-0.0698					0.70 (0.50-0.80)	0.60 (0.50-0.80)
										
Other retinopathy	No	1129	-0.23	-0.0239	0.32	0.87	0.18	0.93	1.00 (0.80-1.00)	0.80 (0.67-1.00)
	Yes	112	-0.29	-0.0370					0.80 (0.67-1.00)	0.67 (0.50-1.00)
										
Eye pressure (mmHg)	< 16	369	-0.25	-0.0215	0.58	0.64	0.031	0.045	1.00 (0.70-1.00)	0.90 (0.67-1.00)
	16- < 20	592	-0.22	-0.0234					1.00 (0.80-1.00)	0.80 (0.67-1.00)
	≥ 20	151	-0.28	-0.0441					1.00 (0.75-1.00)	0.80 (0.60-1.00)

Visual acuity is given as logMAR and decimal acuity. Estimates of logMAR are from a mixed model using all available measurements of visual acuity which come from the best seeing eye in all cases. *p *values are from Wald tests. Median values (interquartile range) of decimal acuity are from the first and the last eye examination

^a ^Testing whether the mean of the level of visual acuity at diabetes diagnosis is different for the two or three levels of the stratification variable

^b ^Testing whether the mean yearly change of visual acuity after diabetes diagnosis is different for the two or three levels of the stratification variable, given the level of the visual acuity at diabetes diagnosis. This test focuses on differences in slope between the linear curves describing changes in visual acuity

^c ^HbA_1c _measured within 90 days of diabetes diagnosis. Reference range: 5.4-7.4

## Results

### Baseline characteristics

At diabetes diagnosis median (IQR) age was 65.5 (56.0-73.6) years, male/female ratio was 1.11 (653/588), and median diagnostic plasma glucose was 13.7 (10.7-17.0) mmol/l. The prevalence (95% confidence interval) of blindness and moderate visual impairment was 0.9 (0.4-1.4) % and 5.4 (4.1-6.7) %, respectively (Table 2). AMD, other non-diabetic retinopathy and, above all, cataract were common. The ophthalmologists estimated that cataract was the most common cause of visual impairment except among the blind (Table 2). At diagnosis the eye doctors reported glaucoma in the best seeing eye in 18 (1.5%) of 1241 patients. 13 had normal visual acuity and 5 had impaired vision.

**Table 2:** Visual acuity at diabetes diagnosis according to age, sex, retinopathy, and cataract

	Visual acuity ^a^			
		
	Normal	Moderately impaired	Blind	All patients
	*n *= 1,163	*n *= 67	*n *= 11	*n *= 1,241
Age (years)				
40- < 60	429 (36.9)	1 (1.5)	0 (0)	430 (34.7)
60- < 70	366 (31.5)	10 (14.9)	1 (9.1)	377 (30.4)
70+	368 (31.6)	56 (83.6)	10 (90.9)	434 (35.0)
				
Sex				
Male	622 (53.5)	28 (41.8)	3 (27.3)	653 (52.6)
Female	541 (46.5)	39 (58.2)	8 (72.7)	588 (47.4)
				
Retinopathy ^a^				
Diabetic retinopathy				
Microaneurysms only	13 (1.1)	0 (0)	1 (9.1)	14 (1.1)
Further diabetic retinopathy	34 (2.9)	2 (3.0)	2 (18.2)	38 (3.1)
Age-related macular degeneration, AMD	117 (10.1)	30 (44.8)	7 (63.6)	154 (12.4)
Other	104 (8.9)	7 (10.5)	1 (9.1)	112 (9.0)
No retinopathy	908 (78.3)	32 (47.8)	1 (9.1)	941 (76.0)
				
Cataract ^a^	278 (24.2)	55 (82.1)	4 (36.4)	337 (27.5)
				
Ophthalmologist's indication of reason for visual impairment ^a, b^				
Retinopathy of all sorts	76 (6.5)	13 (19.4)	5 (45.4)	94 (7.6)
Cataract	171 (14.7)	25 (37.3)	1 (9.1)	197 (15.9)
Other causes	42 (3.6)	6 (9.0)	2 (18.2)	50 (4.0)
Combination of causes	45 (3.9)	20 (29.9)	2 (18.2)	67 (5.4)
None	829 (71.3)	3 (4.5)	1 (9.1)	833 (67.1)

Data are numbers (column-%)

^a ^Refers to best seeing eye

^b ^The ophthalmologists answered this question even for patients who were mildly visually impaired although their visual acuity was normal per definition, i.e. ≥ 0.5

### Change in visual acuity

Among the 807 surviving and re-examined patients, visual acuity generally deteriorated (Table 3). The prevalence (95% confidence interval) of blindness and moderate visual impairment was 2.4 (1.3-3.4) % and 6.7 (5.0-8.4) %, respectively, 5.6 (5.0-6.3) years (median, IQR) after the first eye examination. The 18 new-blind patients in Table 3 represent an incidence (95% confidence interval) of blindness of 40.2 (25.3-63.8) per 10,000 patient-years. Of 18 new-blind patients, 14 were over 70 years. The incidence of moderately impaired vision or worse was 142.3 (110.5-183.2) per 10,000 patient-years among patients with normal sight at diagnosis. Of the 25 patients in Table 3 with moderate visual impairment at baseline, 14 had normal visual acuity 6 years later. Of these 14 patients, 7 had had a cataract operation since the baseline examination and one had had a retinal laser treatment.

**Table 3:** Changes in visual acuity from diabetes diagnosis until 6-year follow up

Visual acuity at diagnosis ^a^	Visual acuity at 6-year follow up ^a^			
		
	Normal	Moderately impaired	Blind	Total
Normal	720 (92.3)	48 (6.2)	12 (1.5)	780 (96.7)
Moderately impaired	14 (56.0)	5 (20.0)	6 (24.0)	25 (3.1)
Blind	0 (0)	1 (50.0)	1 (50.0)	2 (0.2)
Total	734 (90.9)	54 (6.7)	19 (2.4)	807 (100)

Data are numbers (row- and column-%)

^a ^Refers to best seeing eye at diabetes diagnosis and 6-year follow up, respectively

**Figure 2:**
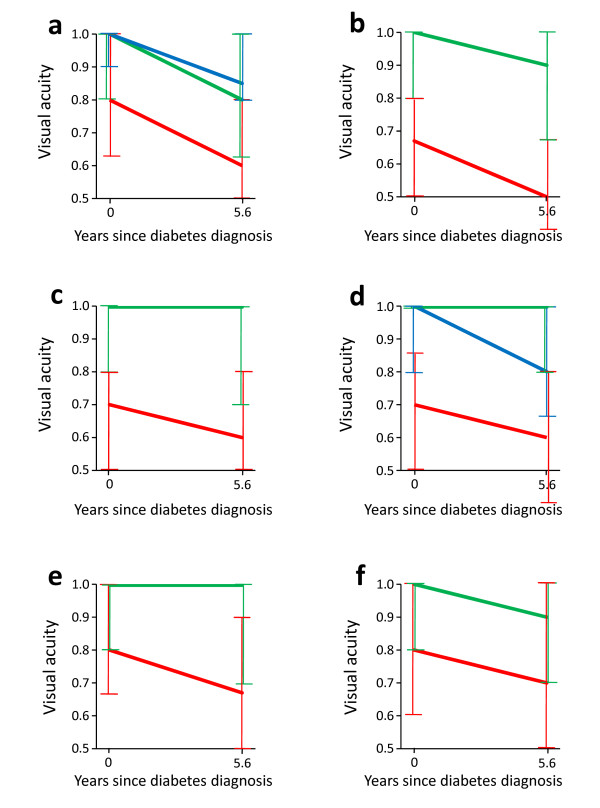
**Vision loss during the first 6 years after diabetes diagnosis according to all statistically significant predictors at diagnosis except fasting triglycerides and self-rated health**. The curves are defined by medians of decimal acuity (interquartile ranges) at diagnosis and 6 years later. **a **Diabetic retinopathy: no retinopathy (green); microaneurysms only (blue); further retinopathy (red). **b **Age-related macular degeneration: no (green); yes (red). **c **Cataract: no (green); yes (red). **d **Age, years: 40- < 60 (green); 60- < 70 (blue); ≥ 70 (red). **e **Living alone: no (green); yes (red). **f **Physical activity: moderate/high (green); low (red).

During the 6 years of follow up, DR had appeared in 11.7% (90/770) of patients without DR at diagnosis (Table 4). At 6-year follow up, DR and AMD were also relatively more common among the visually impaired (Table 5). The influence of eye complications at diagnosis on change in visual acuity over 6 years was investigated in linear mixed models (Table 1). The 112 patients in Table 1 with "other retinopathy" presented with133 retinal pathologies other than DR and AMD: hypertensive retinopathy (n = 43), retinal vasosclerosis (41), drusen (18) and other retinopathy (31). DR (n = 52), AMD (154), and cataract (337) were associated with the level of visual acuity and/or its annual change also in age- and sex-adjusted analyses. These effects are illustrated with median decimal acuity values in Figure 2.

**Table 4:** Prevalence of diabetic retinopathy at diabetes diagnosis and at 6-year follow up

	Diabetic retinopathy at 6-year follow up ^a^					
		
Diabetic retinopathy at diabetes diagnosis ^a^	No diabetic retinopathy	Background retinopathy				
				
		Microaneurysms only	Without maculopathy	With maculopathy	Proliferative retinopathy ^b^	Total
No diabetic retinopathy	680 (88.3)	46 (6.0)	32 (4.2)	10 (1.3)	2 (0.3)	770 (96.0)
						
Background retinopathy						
Microaneurysms only	2 (20.0)	3 (30.0)	4 (40.0)	1 (10.0)	0 (0)	10 (1.2)
Without maculopathy	8 (47.1)	1 (5.9)	3 (17.6)	1 (5.9)	4 (23.5)	17 (2.1)
With maculopathy	0 (0)	1 (20.0)	0 (0)	3 (60.0)	1 (20.0)	5 (0.6)
						
Proliferative retinopathy	0 (0)	0 (0)	0 (0)	0 (0)	0 (0)	0 (0)
						
Total	690 (86.0)	51 (6.4)	39 (4.9)	15 (1.9)	7 (0.9)	802 (100)

Data are numbers (row- and column-%)

^a ^Refers to the eye with the most pronounced diabetic retinopathy at diabetes diagnosis and 6-year follow up, respectively

^b ^Among these, 2 patients with new vessels on the optic disc

**Table 5:** Visual acuity and retinopathy 6 years after diabetes diagnosis

	Visual acuity ^a^			
		
	Normal	Moderately impaired	Blind	All patients
Retinopathy ^a^	*n *= 734	*n *= 54	*n *= 16	*n *= 804
Diabetic retinopathy				
Microaneurysms only	46 (6.3)	5 (9.3)	0 (0)	51 (6.3)
Further diabetic retinopathy ^b^	48 (6.5)	10 (18.5)	3 (18.8)	61 (7.6)
				
Age-related macular degeneration, AMD	65 (8.9)	20 (37.0)	11 (57.9)	96 (11.9)
				
Other retinopathy	44 (6.0)	0 (0)	2 (10.5)	46 (5.7)
				
No retinopathy	541 (73.7)	23 (42.6)	1 (6.3)	565 (70.3)

Data are numbers (column-%)

^a ^Refers to best seeing eye

^b ^Among patients with normal eye sight, impaired eye sight and blindness there are 4, 2 and 1 patient, respectively, with proliferative retinopathy

Similarly, some socio-demographic, clinical, biochemical, and behavioural variables affected the level of visual acuity and/or its annual change over 6 years even when the effect of age and sex was considered (Table 1). All significant effects, except that of self-rated health and triglycerides, are illustrated in Figure 2. The effect of triglycerides was not picked up by the median values. This is because the median figures in Figure 2 emphasize typical developments in visual acuity, while the result of the statistical analysis is necessarily based on logMAR values and expressed as means which are greatly affected by patients with very poor eye sight (Table 1).

When the changes in visual acuity were modelled as piecewise linear models with a breakpoint 3 years after diagnosis, the annual change in logMAR before (0.0268, p < 0.0001) and after this point (0.0231, p = 0.0008) was similar (p = 0.99 for the significance test of the time-logMAR interaction). Accordingly, visual loss during the first years after diabetes diagnosis did not seem to be greater than later on, which supports the underlying assumption in Figure 2 that visual loss follows a linear course.

Adding the identification number of the eye doctors as a random effect to the models in Table 1 did not change the associations with developments in visual acuity. Therefore, it is unlikely that our results are affected by the fact that some patients were examined by the same eye doctor at baseline and follow up, while others were not.

## Discussion

In a population-based cohort of patients newly diagnosed with clinical, often symptomatic type 2 diabetes, 6.3% were visually impaired. Among those patients with reduced sight, 76% had cataract and 58% retinopathy, usually AMD, although many of these eye pathologies are not closely related to diabetes and metabolic control. During the first 6 years after diabetes diagnosis, the incidence of blindness was relatively high, 40 per 10,000 person-years. The baseline predictors of both the level of and speed of progressive visual impairment after diagnosis were AMD, cataract, age at diagnosis, and living alone. The level of visual acuity over 6 years was lower in patients who had a low self-rated health or a sedentary life-style. The rate of the 6-year visual loss increased if the patient had DR or high fasting triglycerides at diabetes diagnosis.

### Strengths and limitations of the study

Our patients are likely to be representative of Danish patients with newly diagnosed, clinical, often symptomatic diabetes in this age group because of the well-defined background population in each general practice, the unchanged inclusion activity during the inclusion period, the small number of primary exclusions, and the acceptable participation in the eye examinations [24]. Furthermore, data from studies including patients with known diabetes may be misleading because of selective survival of patients with a more favourable risk factor profile [29]. Our results, however, cannot be generalised to all countries because of marked disparities in causes of reduced vision across the world [8].

Our patient sample was established in the early 90's and since then both surgical treatment of eye pathologies and pharmacological treatment of hyperglycaemia, hypertension and dyslipidaemia have been intensified, as has screening for diabetes. These initiatives to improve diabetes care and to identify patients earlier in the natural history of diabetes have probably decreased the variability of measures of treatment quality, so if a similar follow up study were to be made today, the associations between variables would perhaps be harder to identify. There is, however, no reason to suppose that the causal patterns underlying the associations that we have identified would be substantially different.

By presenting results from the best seeing eye only, we are underestimating the prevalence of eye pathologies in this patient group. This is because our main purpose is to estimate the change in visual acuity and its predictors, and eye pathologies are important in this study only insofar they are predictors of visual loss. In most studies the main pathology in the better eye is considered the cause of visual impairment [6,7]. We analysed predictors of vision impairment prospectively. Furthermore, to improve statistical strength we chose to analyse visual acuity as a continuous outcome.

Our ability to detect a true clinical change in visual acuity is inversely related to the measurement error, i.e. the test-retest variability, of the test used [30,31]. Unlike logMAR charts, the Snellen chart, which we had to rely on in this nationwide primary care study, has a large-scale increment resulting in a relatively high measurement error. Another factor contributing to the test-retest variability was differing routines for taking account of visual field loss [32]. The lower precision of the outcome, visual acuity, will however only tend to lessen the strength of the association between the outcome and a predictor.

On the other hand, the true incidence of vision impairment may be underestimated if those patients who missed an eye examination experienced a relatively rapidly declining vision. However there is no reason to suppose that the possible imprecision and error introduced were associated with any of the possible predictors of visual loss that were examined. Comparison of visual acuity between studies, even when categorised, is in any case not feasible [31], and our prevalence and incidence figures for visual impairment should be interpreted in this light.

Almost all Danish ophthalmologists contributed to the study increasing the inter-rater variability, and their screening by funduscopy may have overlooked 10-40% of sight-threatening eye disease [33]. Such measurement errors in the predictor variables will tend to reduce a true association between e.g. an eye pathology and the outcome, i.e. visual acuity, but it does not invalidate the associations that we actually find. It can be assumed that the detection rate for eye disease was higher in patients with low visual acuity. This may have biased the cross-sectional associations between eye disease and visual acuity in Table 2 and 5, but it does not to the same extent compromise the estimation of the predictive power of the eye pathologies at diagnosis for the change in visual acuity during the following 6 years. Therefore, our non-standardized estimation of eye pathologies at diabetes diagnosis only diminishes our ability to detect an effect of these variables on changes in visual acuity.

### Comparison with existing literature

#### Predictors of 6-year visual loss

In Table 2 and in most studies [3-9,34-36] the main eye pathology is assigned as the cause of visual impairment. We analysed prospectively 26 possible ocular and non-ocular predictors of vision loss (Table 1). It is striking that, besides age and triglycerides, only DR, AMD, and cataract, many of which are surgically modifiable, were associated with declining vision over 6 years. This was observed even though the measurement error in the estimation of these eye pathologies is considered to be greater than for many of the other possible predictors. In follow up studies, the association between baseline DR or AMD and later impaired vision is well documented [14-16,19], and AMD may cause deterioration in visual acuity earlier in diabetic patients than in non-diabetic people though the prevalence of AMD does not seem to differ markedly between the two groups [17]. Furthermore, diabetic subjects have a 2 to 4 times greater risk of developing cataract than non-diabetic people [18].

As our way of collecting information about glaucoma may underestimate the true prevalence of glaucoma in our patients, we were not able to analyse the predictive effect of glaucoma for visual loss. The possible importance of eye pressure for the change in eye sight over 6 years is, however, indicated by the non-significant tendency reported in Table 1.

Only a few prospective studies have assessed non-ocular predictors of visual loss other than age and sex [14,16,19-23]. The only study including more than a few possible predictors used a subjective measure of visual dysfunction [14], while the most comprehensive study until now using measured visual acuity examined the effect of HbA1c, blood pressures, proteinuria and smoking as well as age and sex [19]. Among many candidate predictors we found only relatively high age, living alone and high triglycerides to be associated with worsening of visual acuity over 6 years, while high age, living alone, low self-rated health, and low level of physical activity were associated with a low level of visual acuity. Presumably the three last-mentioned relations are cases of reverse causation where poor vision affects living conditions. Marital status has similarly been found to predict vision loss in men with older-onset diabetes [20], but the association was reversed in a study of patients with advanced DR [21].

In UKPDS the incidence of visual impairment was slightly lower in the tight vs. the less tight blood pressure control group [22]. Similarly, blood pressure, HbA1c and proteinuria have been shown to be indicative of visual loss in follow up studies [16,19,23], but none of these non-ocular patient variables was associated with visual loss in the present study. This could be due to measurement error and above all regression dilution bias [37], which is particularly relevant for biochemical and clinical variables in the dysmetabolic state of newly diagnosed clinical diabetes. In studies including patients with known diabetes [16,19,23], the measured risk factor levels are supposedly closer to an average steady state level, a kind of set point which is typical for the patient in question. In the present study, however, high level of triglycerides, which has been identified as a risk factor for proliferative DR [38], was a significant predictor of declining vision. In line with this finding, the FIELD study showed a promising reduction in the need for laser treatment for DR after treatment with fenofibrate but this did not affect worsening of visual acuity [39]. It is possible that DR mediates the effect of triglycerides on visual acuity in a slow, progressive pathophysiological process. The strong counter-intuitive inverse univariate relation between smoking and visual acuity disappeared after age and sex adjustment (Table 1) while a similar association between smoking and DR persisted after adjustment in UKPDS [40].

#### Prevalence and incidence of blindness and moderate visual impairment

In population-based studies of patients with known type 2 diabetes the prevalence of blindness is between 1% and 3% [6,7,34,41-43], lowest in populations offered regular eye screening. The prevalence increases markedly with diabetes duration [14,23,42,43], in the present study from 0.9% at diagnosis to 2.4% six years later (Table 3).

With the gradual implementation of systematic eye screening the incidence of blindness among diabetic patients has declined [44]. In the Nordic countries the incidence per 10,000 person-years has declined from the range of 200-500 [15,45] in the early 1980s to more recent figures of about 15 [23,46] among persons with known type 2 diabetes. Our patient sample included many old patients and both the prevalence [2,4,41] and the incidence [14,16,19] of visual impairment increases curvelinearly with age as in the background population [2,36,47]. This may partially explain why the incidence rate was as high as 40 per 10,000 person-years in the present study.

In studies of patients with known type 2 diabetes, which are to some extent population-based, the prevalence of 7-11% for moderate visual impairment [22,34,42,43] is similar to the 6.7% observed 6 years after diagnosis in the present study. Of the 54 patients with moderate visual impairment at this point, however, 48 had normal vision at diagnosis, but the prevalence rate increased only modestly because of the over-mortality of patients with low vision [48].

### Implications for clinical practice and future research

It is evident that even in Denmark where patients can refer themselves to free eye examinations, treatable eye pathologies such as DR and cataract predict further visual loss. Our results underline the importance of eliminating barriers to efficient eye care by facilitating access to eye examination, increasing the understanding of patients and primary care practitioners of the need for regular screening and early surgical treatment, and, in some countries, addressing patients' financial burdens [49]. It should be easy to motivate a thoroughly informed patient to have regular eye examinations as many patients fear visual loss as the worst consequence of diabetes [10,13]. The fact that almost all Danish primary care eye doctors participated in the present study demonstrates the commitment of ophthalmologists to preventive diabetes care.

In primary care, future intervention studies to reduce visual impairment in patients with diabetes may be designed primarily to overcome barriers to effective eye care, and such trials should preferably distinguish between the effect on visual acuity of changes in biological age on retina and of the interventions targeting treatable eye pathologies.

## Conclusions

To conclude, severely reduced sight is a very real challenge for patients with newly diagnosed clinical, often symptomatic type 2 diabetes. During the first 6 years after diabetes diagnosis visual acuity deteriorates considerably, and this visual loss depends primarily on age and the presence of AMD, DR and cataract at diagnosis. Patients newly diagnosed with clinical type 2 diabetes should be made aware that there is an inevitable age-related decline in sight but that further vision loss associated with diabetes is largely preventable through diligent ophthalmological follow up and surgical intervention.

## Competing interests

The authors declare that they have no competing interests.

## Authors' contributions

NDFO conceived of and designed the study, carried out data collection, and drafted the manuscript. NDFO and NVN designed and coded the eye questionnaires. VS did the statistical analyses. NDFO, VS, GJA and NVN participated in the interpretation of data and in the revisions of the manuscript. All authors have read and approved the final manuscript.

## Pre-publication history

The pre-publication history for this paper can be accessed here:

http://www.biomedcentral.com/1471-2458/11/80/prepub
